# Within-patient horizontal transfer of pOXA-48 from a hypervirulent *Klebsiella pneumoniae* SL218 to *Serratia marcescens* following spread of the *K. pneumoniae* isolate among hospitalised patients, Denmark, 2021 

**DOI:** 10.2807/1560-7917.ES.2023.28.17.2300196

**Published:** 2023-04-27

**Authors:** Karen Leth Nielsen, Marc Sørensen, Frederik Boëtius Hertz, Maria Anna Misiakou, Henrik Hasman, Susanne Häussler, Marie Helleberg, Kristian Schønning

**Affiliations:** 1 Department of Clinical Microbiology, Copenhagen University Hospital - Rigshospitalet, Copenhagen, Denmark; 2 Department of Infectious Diseases, Copenhagen University Hospital - Rigshospitalet, Copenhagen, Denmark; 3 Department of Thoracic Anaesthesiology, Copenhagen University Hospital - Rigshospitalet, Copenhagen, Denmark; 4 Department of Genomic Medicine, Copenhagen University Hospital - Rigshospitalet, Copenhagen, Denmark; 5 Bacteria, Parasites and Fungi, Statens Serum Institut, Copenhagen, Denmark; 6 Department Molecular Bacteriology, Helmholtz Centre for Infection Research, Braunschweig, Germany; 7 Twincore, Centre for Experimental and Clinical Infection Research, a joined venture of the Helmholtz Centre for Infection Research and the Hannover Medical School, Hannover, Germany; 8 Center of Excellence for Health, Immunity and Infections, Copenhagen University Hospital - Rigshospitalet, Copenhagen, Denmark; 9 Department of Clinical Medicine, Faculty of Health and Medical Sciences, University of Copenhagen, Copenhagen, Denmark

**Keywords:** Hospital-acquired infections, within-patient plasmid transfer, hypervirulent *K. pneumoniae*, antimicrobial resistance, OXA-48

## Abstract

A hypervirulent *Klebsiella pneumoniae* SL218 (ST23-KL57), phylogenetically distinct from the classical hypervirulent SL23 (ST23-KL1) lineage, was transmitted between hospitalised patients in Denmark in 2021. The isolate carried a hybrid resistance and virulence plasmid containing *bla*_NDM-1_ and a plasmid containing *bla*_OXA-48_ (pOXA-48); the latter plasmid was horizontally transferred within-patient to *Serratia marcescens*. The convergence of drug resistance and virulence factors in single plasmids and in different lineages of *K. pneumoniae* is concerning and requires surveillance.

*Klebsiella pneumoniae* sequence type (ST)23 is considered associated with hypervirulence [1,2]. However, ST23 defined by classical 7-loci multilocus sequence typing (MLST) includes two distinct sublineages (SL), SL23 comprising ST23 strains with capsular locus (KL) 1 associated with hypervirulence and the phylogenetically distant SL218 comprising strains with KL57. 

Here, we describe the nosocomial transmission of a novel hypervirulent and multidrug-resistant *K. pneumoniae* SL218 isolate carrying a *bla*_NDM-1_ and a *bla*_OXA-48_ β-lactamase among co-patients isolated in a Danish university hospital in 2021. We characterise the plasmid structure of the isolates and describe within-patient horizontal transfer of a plasmid carrying *bla*_OXA-48_ from the hypervirulent *K. pneumoniae* SL218 to *Serratia marcescens*.

## Clinical case description

Patient A with laboratory-confirmed COVID-19 was transferred to a Danish university hospital from a nearby hospital due to severe acute respiratory distress syndrome and need for extracorporeal membrane oxygenation (ECMO). The ECMO was initiated upon admission.

At this hospital, during the COVID-19 pandemic, patients diagnosed with COVID-19 were cohort-isolated in the intensive care unit if isolation in a single room was not possible. Within the isolation cohort, separate staff was allocated to each patient. Patient A was cohort-isolated with patients who had been hospitalised outside of Denmark. All patients hospitalised for more than 24 h outside of Denmark within 6 months before admission are screened for CPO carriage and close contact patients are similarly screened. Microbiological cultures of rectal screening swabs from Patient A identified carriage of *K. pneumoniae* carrying *bla*_OXA-48_ and *bla*_NDM-1_ on day 8 upon admission. Patient A was screened because a co-patient was found positive for carbapenemase-producing bacteria. The two patients were subsequently isolated in separate rooms as cohort isolation is not used for patients with carbapenemase-producing organisms.

**Figure 1:**
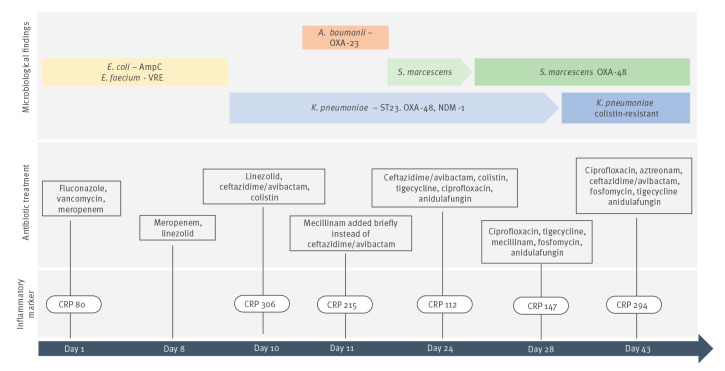
Treatment timeline for a COVID-19 patient with multiple bacterial infections, Denmark, 2021

BAL: bronchoalveolar lavage; CRP: C-reactive protein; ST: sequence type; VRE: vancomycin-resistant *Enterococcus faecium*.

Patient A was treated with ECMO for overall 46 days during which laboratory testing revealed changing signs of inflammation, including elevated levels of white blood cells, C-reactive protein, and procalcitonin. *Klebsiella pneumoniae, S. marcescens, Acinetobacter baumannii*, *Enterococcus faecium* and *Candida* spp. were cultured repeatedly from different sites, and the patient was treated appropriately with adjusted antibiotic regimens (Figure 1). Further details on treatment timeline and inflammatory markers can be found in Supplementary Table S1. After 2 months of continued ECMO treatment, the patient developed a cerebral haemorrhage and further treatment was ceased.

Here we list the bacterial isolates (site of infection): *Escherichia coli* AmpC (urinary tract), VRE (urinary tract). *Klebsiella pneumoniae* SL218, OXA-48, NDM-1 (blood and BAL) + *Acinetobacter baumanii* OXA23 (tracheal aspirate). *Serratia marcescens* (BAL). *S. marcescens* (BAL) +* S. marcescens pOXA-48* (tracheal aspirate). *K. pneumoniae *colistin-resistant (tracheal aspirate). CRP values are given as mg/L (normal range: < 10mg/L).

## Phenotypic and molecular characterisation of *Klebsiella pneumoniae* and *Serratia marscesens* isolates

Antimicrobial susceptibility testing was performed using European Committee on Antimicrobial Susceptibility Testing (EUCAST) guidelines and expert rules [3]. Susceptibility testing was performed by disk diffusion and minimal inhibitory concentrations (MIC) using gradient tests, except for colistin where microbroth MIC determination was done. Phenotypic susceptibility is summarised in Table 1. In addition to the agents shown in Table 1, the *K. pneumoniae *isolate was tested for trimethoprim/sulfametoxazol (MIC: 32 mg/L), tobramycin (R: 6 mm) and tigecycline (MIC: 2 mg/L). The initial *K. pneumoniae* isolate was susceptible to colistin (MIC: 0.25 mg/L) but isolates after day 27 had developed resistance (MIC: 16 mg/L). Synergy was observed for the combination of ceftazidime/avibactam and aztreonam using gradient tests. 

**Table 1:** Phenotypic susceptibility determination of *Klebsiella pneumoniae* and *Serratia marcescens* isolates from a patient with multiple bacterial infections, Denmark, 2021

Antibiotic^a^	TZP (zone)	MEC (zone, MIC)	CRO (zone)	CAZ (zone)	CFDC (zone)	CZA (MIC)	ATM (zone, MIC)	MEM (zone)	CIP (zone)	FOS (MIC)
*Klebsiella pneumoniae* OXA-48, NDM-1	R (6 mm)	S-> R (15–> 6 mm, 4 mg/L)	R (6 mm)	R (6 mm)	R (6 mm)	R^b^	R^b ^ (6 mm, 256 mg/L)	R (6 mm)	R (6 mm)	S (16–24 mg/L)
*Serratia marscesens*	S (25 mm)	MIC 2 mg/L	NT^c^	S (28 mm)	NT	S (0.25 mg/L)	S (30 mm)	S (30 mm)	S (30 mm)	S (16 mg/L)
*Serratia marscesens* OXA-48	R (6 mm)	MIC 2 mg/L	NT^c^	S (27 mm)	NT	S (0.5 mg/L)	S (30 mm)	I (17 mm)	S (28 mm)	NT

ATM: aztreonam; CAZ: ceftazidime; CFDC: cefiderocol; CIP: ciprofloxacin; CRO: ceftriaxone; CZA: ceftazidime-avibactam; FOS: fosfomycin; MEC: mecillinam; MEM: meropenem; MIC: minimal inhibitory concentration; NT: not tested; TZP: piperacillin/tazobactam; zone: zone diameter.

^a^ Phenotypic susceptibility testing was performed according to EUCAST guidelines [3].

^b^ Synergy was observed for the combination of ceftazidime/avibactam and aztreonam using gradient tests.

^c^ Not used due to inducible AmpC.

For elaborated laboratory methods, please see the supplementary material. We isolated *K. pneumoniae* from two patients during their hospitalisation. Both *K. pneumoniae* isolates displayed a hypermucoid phenotype (positive string test). A picture demonstrating this test result is appended to this publication in Supplementary Figure S1. 

Whole genome sequencing revealed that the isolates belonged to SL218 (scgST-34342), the capsule type inferred from Kleborate (https://github.com/klebgenomics/Kleborate; [4]) was KL57, and SNP analysis performed with BacDist (https://github.com/MigleSur/BacDist; [5]), reference genome: GCF_006364295.1_ASM636429v1) disclosed that the two isolates were identical, indicating transmission between the patients during their cohort isolation. The *K. pneumoniae* SL218 isolate from patient A was subjected to Oxford Nanopore Technology sequencing, and UniCycler hybrid assembly resulted in six circular contigs: a chromosome of 5,165,659 bp and five plasmids of various sizes (247,143 bp, 162,873 bp, 62,722 bp, 4,429 bp and 2,058 bp) (Table 2). The chromosome contained the yersiniabactin virulence module within an ICE*Kp3* element integrated into the third tRNA-*Asn* site [6]. This module contains virulence factors yersiniabactin (*ybt, irp2* and *irp1*) and yersiniabactin uptake receptor (*fyuA*). The large 247,143 bp plasmid encoded aerobactin cluster (*iucB, iucC, iucD* and *iutA*), as well as regulators of extracapsular polysaccharide synthesis (*rmpA, rmpA2* and *rmpC*). The isolates lacked the salmochelin and colibactin gene loci. The resulting Kleborate virulence score was 4. The isolates contained numerous antimicrobial resistance genes including *bla*_NDM-1_, *bla*_OXA-48_, and *bla*_CTX-M-15_ distributed on the replicons as detailed in Table 2.

**Table 2:** Replicons, genotypic antimicrobial resistance and virulence genes identified in *Klebsiella pneumoniae* SL218 and *Serratia marscesens* isolates from a patient with multiple bacterial infections, Denmark, 2021

	Size (bp); replicon	Antimicrobial resistance genes			Virulence^a^
		β-lactams	Aminoglycosides	Other	
*Klebsiella pneumoniae*					
Chromosome	5,165,647	*bla*_SHV-1_	None	*oqx*AB, *fos*A	*ybt*AEPQSTUX, *iut*A, *irp*1,2, *fyu*A
Plasmid #1	247,125; repB	*bla*_NDM-1_	*aph*(3’)-VI	*qnr*S, *sul*1, *sul*2, *dfr*A5, *mph*(E), *msr*(E), *mph(A)*	*iuc*ABCD, *rmp*A, *rmp*A2
Plasmid #2	162,824; IncFIB, IncFII	*bla*_CTX-M-15_	*aac* [1]-IIa, *aac*(6’)-Ib-cr, *rmt*F	*cat*A1, *qnr*B	None
Plasmid #3	62,722; IncL	*bla*_OXA-48_	None		
Plasmid #4	4,429; NT	None			
Plasmid #5	2,058; ColpVC	None			
*Serratia marcescens *					
Chromosome	5,050,061^b^	*bla*_SRT-2_	*aac*(6')-Ic	*oqx*B, *qnr*E1, *tet*(41)	None
Plasmid #1	76,466; NT	None			
Plasmid #2	94,803; NT	None			
*Serratia marcescens* pOXA-48					
Chromosome	5,050,233^b^	*bla*_SRT-2_	*aac*(6')-Ic	*oqx*B, *qnr*E1, *tet*(41)	None
Plasmid #1	76,466; NT	None			
Plasmid #2	94,803; NT	None			
Plasmid #3	63,589; IncL	*bla*_OXA-48_	None		
Plasmid #4	2,058; ColpVC	None			

NT: non-typeable.

^a^ Virulence factors of *K. pneumoniae* identified with Kleborate [4]*.*

^b^ Represents incomplete assembly of chromosome.

**Figure 2:**
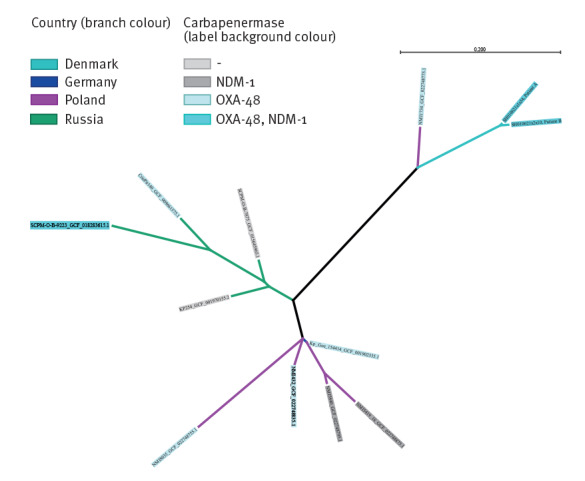
Phylogenetic context of *Klebsiella pneumoniae* isolated in Denmark, 2021 (n = 2)

The isolate was closely related to the SL218 isolate Kp_Goe_154414 (scgST-3381), which was detected in Göttingen, Germany in 2014 [7] (BioSample: SAMN5412805; CP018337) as well as isolates from Poland and Russia [8], with the closest phylogenetic relationship to *bla_OXA-48_*-carrying SL218 isolate NMI1734 (scgST-16235) identified in Poland (Figure 2) [9].

Phylogenetic comparison with Parsnp and Harvest suite to illustrate genetic distance between *K. pneumoniae* of this study in the context of SL218 isolates identified in Russia and later France and Finland from a risk assessment by the European Centre for Disease Prevention and Control [23] and available in GenBank (Accession numbers SRR7181964 and SRS7484649) and isolates belonging to SL218 and carrying OXA-48, NDM-1 or both, described in recent work [9]. The SL218 isolates of the present study are most closely related to NMI1734 (48–50 SNPs) isolated in Poland and carrying *bla*_OXA-48_. The scalebar represents substitutions per site, the maximum single nucleotide polymorphism (SNP) distance across tree is 159 SNPs.

## Hybrid virulence and resistance plasmid of *Klebsiella pneumoniae*

**Figure 3:**
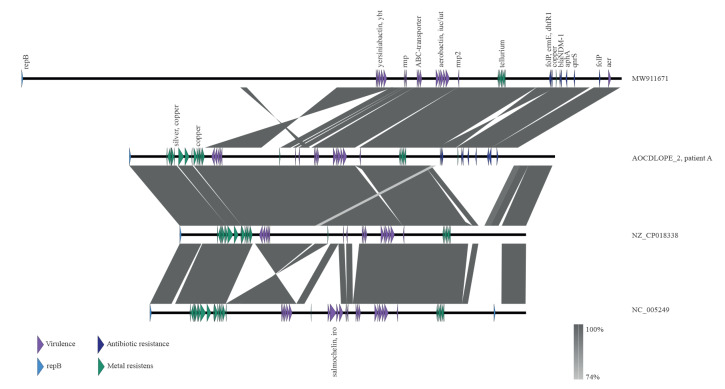
Synteny comparison of the 247,143 bp repB-type hybrid virulence and resistance plasmid of a *Klebsiella pneumoniae* patient isolate, Denmark, 2021, illustrated with related virulence and resistance plasmids

The large repB-type plasmid of 247,143 bp was closely related to the 202,175 bp virulence plasmid from Kp_Goe_154414 (CP018338). Both plasmids had regions homologous to the classical hypervirulence plasmid pLVPK (219,385 bp (NC005249) [10]) but compared with this, both plasmids contained a 21,363 bp deletion that included the salmochelin (*iro*BCDN) locus present in pLVPK (Figure 3). Compared with pLVPK, the repB plasmid of the *K. pneumoniae* SL218 isolate also contained a 48,549 bp insertion containing multiple antimicrobial resistance genes (Figure 3; Table 2), among which *bla*_NDM-1_ was identified in a Tn125 remnant. This multidrug resistance region is highly homologous to similar regions described in hybrid virulence and resistance plasmids that occur for example in *K. pneumoniae* SL147 (MW911671; 99% query coverage and 100% nucleotide identity) and SL395 (MW911666; 100% query coverage and 100% nucleotide identity) reported from Russia [11] (Figure 3).

From top: Hybrid virulence and resistance IncFIB/IncHI1B plasmid from SL147 *K. pneumoniae* from Russia (350,403 bp; MW911671); repB plasmid SL218 *K. pneumoniae* from patient A (247,125 bp; AOCDLOPE_2); 202,175 bp repB plasmid from SL218 isolate Kp_Goe_154414 from Germany (202,175 bp; CP018338); and SL86 *K. pneumoniae* CG43 large virulence repB/IncH1B plasmid pLVPK (219,385 bp; NC_005249). Figure created with Easyfig v. 2.2.5 and finished with Adobe Illustrator.

The *bla*_OXA-48_ gene was located within a Tn1999 element on a separate IncL plasmid of 62,722bp (pOXA-48). This plasmid is highly homologous to other OXA-48 encoding plasmids prevalent in Europe (JN626286) [12].

## In vivo horizontal transfer of pOXA-48 to *Serratia marcescens*

In addition, two *S. marcescens* strains were consecutively isolated from patient A. The second *S. marcescens* isolate was recovered 5 days after the first isolate and, unlike the first isolate, exhibited resistance to piperacillin/tazobactam and diminished susceptibility to meropenem (Table 1). Comparison of the genomes with BacDist considering 88% of the genome (reference genome GCF_000513215.1_DB11), uncovered only a single SNP difference between the two isolates. However, the second *S. marcescens* isolate had acquired two plasmids, a 63,589 bp IncL plasmid containing *bla*_OXA-48 _and a 2,058 bp ColpVC plasmid. The 63,589 bp IncL plasmid was identical to the IncL pOXA-48 plasmid from *K. pneumoniae* except that it had acquired an additional insertion sequence element. The 2,058 bp ColpVC plasmids of *S. marcescens* and *K. pneumoniae* were identical and appeared to have been mobilised with the pOXA-48 plasmid. We make available a detailed illustration of which genes were associated to the two plasmids in Supplementary Figure S2. IncL pOXA-48 plasmids are promiscuously transmitted among Enterobacterales, as exemplified in an outbreak of *bla*_OXA-48_-carrying *K. pneumoniae* in a Dutch hospital involving 118 patients [8]. In 55 of the patients, more than one species of Enterobacterales harboured the pOXA-48, including *S. marcescens*.

## Discussion

Recombination contributes considerably to evolution and diversification of *K. pneumoniae* [1,13,14], and classical 7-locus MLST lacks discriminatory power to accurately capture phylogenetic lineages. As a result, a substantial fraction of classical 7-locus sequence types (ST) are polyphyletic, i.e. a single ST comprises separate phylogenetic lineages [13]. Using a 629-locus core genome MLST (scgMLST) scheme, it is possible to define sublineages (SL) that more accurately reflect phylogeny, i.e. most SL are monophyletic, containing exclusively a single phylogenetic lineage [13]. Certain sublineages, such as SL11, SL15, SL101 and SL147 are globally distributed, often associated with horizontally acquired antimicrobial resistance genes and frequently detected in nosocomial outbreaks [15,16]. Other sublineages (e.g. SL23 or SL65) are associated with hypervirulence but not resistance [17] and are the causative agents of community-acquired invasive infections such as liver abscess, endophthalmitis and meningitis. They are characterised by the expression of virulence factors, most which are encoded on virulence plasmids (e.g. pK2044 and pLVPK) [18]. 

Classical 7-loci MLST classifies both SL23 and SL218 to ST23, however, SL218 (ST23/KL57) isolates are progeny of a recombination event between ST218 and SL395 (ST395) lineages and are phylogenetically not closely related to SL23 isolates [19]. Although hypervirulence and multidrug resistance seemingly appeared in distinct clonal lineages [1], convergent evolution occurs when multidrug-resistant lineages acquire virulence factors or, conversely, hypervirulent lineages acquire antimicrobial resistance genes. Examples of the former are KPC-2-producing *K. pneumoniae* SL11 isolates identified in China, which had additionally acquired a pLVPK-like virulence plasmid [20], and NDM-1-producing *K. pneumoniae* SL147 isolates carrying a hybrid multidrug resistance-virulence plasmid detected in Tuscany, Italy [21]. Examples of the latter include hypervirulent *K. pneumoniae* SL23 isolates, whose genomes encode a *bla*_OXA-48_ observed in Ireland and *K. pneumoniae* SL218 isolates producing NDM-1 or OXA-48, respectively observed in Finland and France [22,23]. The hypermucoid SL218 isolate described here is an additional example of convergence of virulence factors and antimicrobial resistance genes within the same sublineage.

## Conclusion

The carriage of a hybrid resistance and virulence plasmid containing key virulence factors aerobactin (*iut*) and *rmp*AC and a multidrug resistance region carrying *bla*_NDM‑1_ may be especially concerning. In addition, the isolate contained a *bla*_OXA-48_-encoding IncL plasmid capable of dispersion in *Enterobacterales*. The isolate was capable of nosocomial transmission and of in vivo transmission of the IncL plasmid to *S. marscescens*. Related isolates have previously been detected in Germany, Poland and Russia, indicating that this clone might be established and endemic in at least parts of Europe. The detection of hybrid virulence and multidrug resistance plasmids in *K. pneumoniae* SL218 is concerning and requires continued surveillance.

## Supplementary Data

388000 Supplement
