# Supplementary material for: Within-patient horizontal transfer of pOXA-48 from a hypervirulent Klebsiella pneumoniae SL218 to Serratia marcescens following spread of the K. pneumoniae isolate among hospitalised patients, Denmark, 2021
Source: Euro Surveill. 2023 Apr 27;28(17):2300196. doi: 10.2807/1560-7917.ES.2023.28.17.2300196 (PMC10283472; doi:10.2807/1560-7917.ES.2023.28.17.2300196)
Supplement: Supplement [file 23-00196_HERTZ_Supplement.pdf]

This supplementary material is hosted by *Eurosurveillance* as supporting information alongside the article '**Within-patient horizontal transfer of pOXA-48 from a hypervirulent *Klebsiella pneumoniae* SL218 to *Serratia marcescens* following spread of the *K. pneumoniae* isolate among hospitalised patients, Denmark, 2021**', on behalf of the authors, who remain responsible for the accuracy and appropriateness of the content. The same standards for ethics, copyright, attributions, and permissions as for the article apply. Supplements are not edited by *Eurosurveillance* and the journal is not responsible for the maintenance of any links or email addresses provided therein.

Supplement S1. Supplementary methods

Genotyping and whole genome sequencing

In brief, the isolates were whole-genome sequenced using paired-end reads on an Illumina Nextseq platform (2x150bp).DNA was purified with Qiagen blood and tissue DNA extraction kit, followed by library preparation following Hackflex protocol(1). In addition to this, high molecular weight DNA was prepared with Nanobind UMHW protocol followed by library preparation with Rapid barcoding kit (Nanopore) and R9.4.1 flow cell before sequencing on an Oxford Nanopore Technology (ONT) Mk1C sequencing device for generation of long reads. The Illumina genomes were assembled with Spades and annotated with Prokka v.1.12(2). Unicycler (3) was applied for hybrid assembly to close the genome fully and visualized with Bandage. Resistance genes were identified with Abricate with Resfinder database. MLST types were identified with Tsemann/MLST (https://github.com/tseemann/mlst) applying the PubMLST database(4). Plasmid inc-groups were identified with Plasmidfinder (5,6). The single nucleotide polymorphism (SNP) distance

between the two Serratia and two Klebsiella, respectively, was identified with BacDist (https://github.com/MigleSur/BacDist) using raw reads of the two isolates as well as Serratia reference genome GCF_000513215.1_DB11 and *Klebsiella pneumoniae* reference GCF_006364295.1_ASM636429v1, respectively. The accessory genome of the resistant and susceptible *Serratia Marcescens* was compared using GenAPI (https://github.com/MigleSur/GenAPI) and confirmed by read mapping in Geneious Prime vR.9. Additionally, reads from the *K. pneumoniae* carrying *bla*$_{OXA-48}$ was mapped towards the two *S. Marcescens* isolates in Geneious Prime. Further characterization of the *Klebsiella pneumoniae* was performed using Kleborate and Kaptive (7). To determine close relatives of already published hypervirulent isolates we performed SNP analyses with Parsnp and Harvest suite(8) to determine the SNP difference between the present isolate and ST23/KL57 isolates identified in Russia and later France and Finland (GenBank accession SRR7181964 and SRS7484649) available from ECDC risk assessment as well as isolates belonging to ST23/KL57 and carrying *bla*$_{OXA-48}$, *bla*$_{NDM-1}$ or both, described in recent work by Biedrycka et al. 2022 (9).

Supplementary Figure S1.

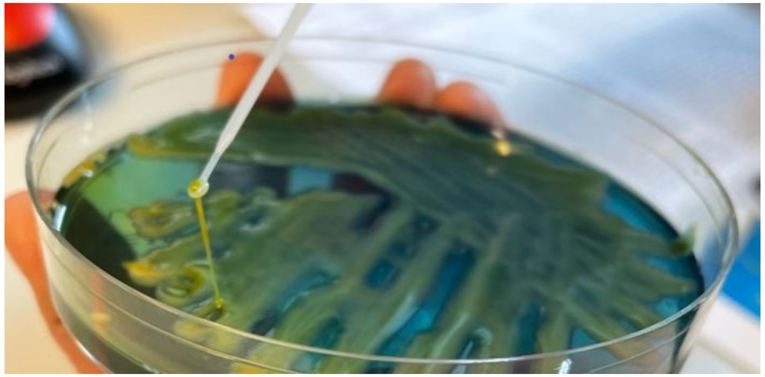

Klebsiella pneumoniae displaying a hypermucoid phenotype (positive string test).

Supplementary Table S1. Timeline of antimicrobial treatment, inflammatory markers and bacteriological findings of a patient with multiple bacterial infections, Denmark.

Day 1	Day 9	Day 8-10	Day 11	Day 15	Day 21	Day 24	Day 26	Day 28	Day 43
Antibiotic treatment									
Fluconazol, meropenem, vancomycin	Meropenem	Meropenem, linezolid	Linezolid, ceftazidime/avibactam, colistin, mecillinam (latter added briefly))	Tigecycline and anidulafungin added	Colistin, tigecycline, anidulafungin, meropenem	Ceftazidime/avibactam, colistin, tigecycline, ciprofloxacin, anidulafungin		Ciprofloxacin, tigecycline, mecillinam, fosfomycin, anidulafungin	Ciprofloxacin, aztreonam, ceftazidime/avibactam, fosfomycin, tigecycline, anidulafungin
Inflammatory markers									
CRP 80 mg/L, LEU 9.8 × 10^9/L, PCT 0.08 µg/L	No change	CRP 215 mg/L, LEU 8.0 × 10^9/L, PCT 0.17 µg/L	CRP 306 mg/L, LEU 5.4 × 10^9/L, PCT 0.92 µg/L	CRP 205 mg/L, LEU 4.5 × 10^9/L, PCT 0.5 µg/L	CRP 92 mg/L, LEU 8.0 × 10^9/L,l PCT 0.1 µg/L	CRP 112 mg/L, LEU 10.9 × 10^9/L, PCT 0.16 µg/L		CRP 147 mg/L, LEU 13.0 x 10^9/L	
Microbiological findings (site of infection)									
Escherichia coli AmpC (urinary tract), VRE (urinary tract)		*Klebsiella pneumoniae* SL218, OXA-48, NDM-1 (blood and BAL) + *Acinetobacter baumanii* OXA23 (tracheal aspirate)	*Serratia marcescens* (BAL)		*S. marcescens* (BAL) + *S. marcescens pOXA-48* (tracheal aspirate)		*K. pneumoniae* colistin: R (tracheal aspirate)		

BAL: bronchoalveolar lavage; CRP: C-reactive protein; LEU: leukocytes; PCT: procalcitonin; R: resistant; VRE: vancomycin-resistant *Enterococcus faecium*.

Supplementary figure S2

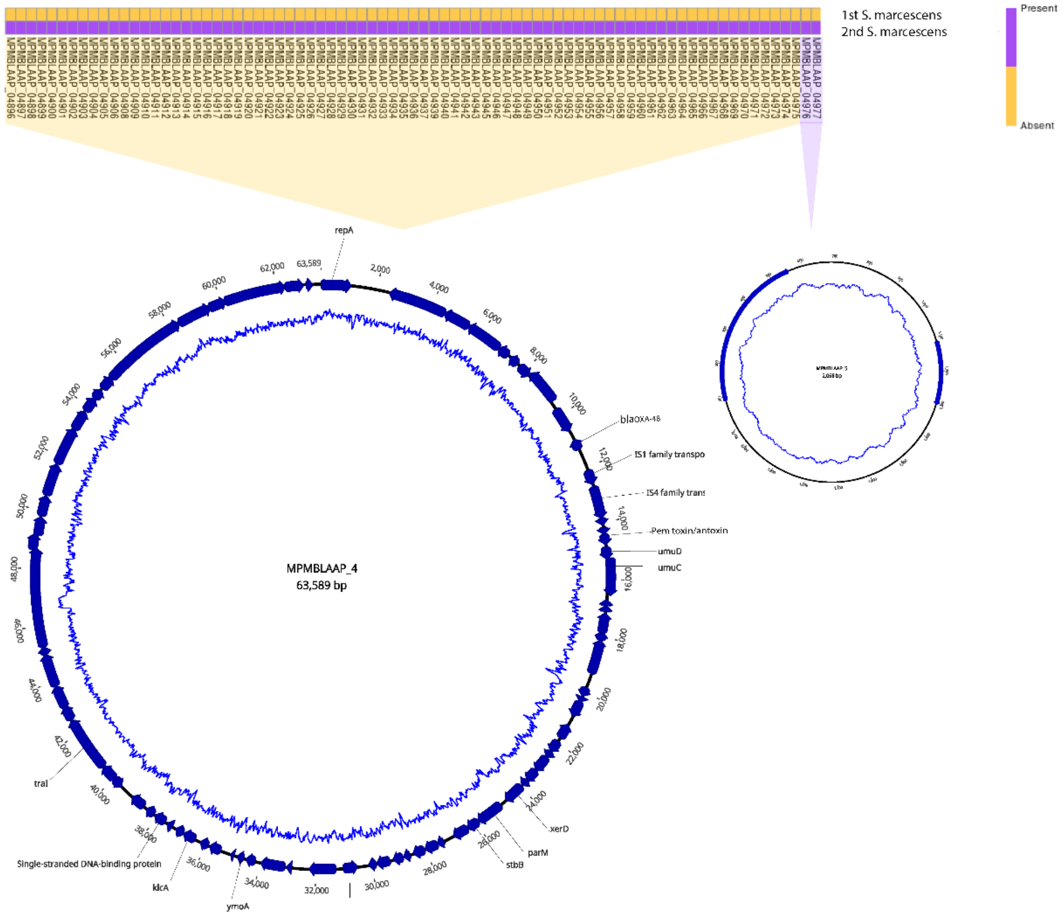

Supplementary Figure S2. Heatmap illustrating unique genes present in the 1st and 2nd *S. marcescens*, respectively. The locus tags all identify genes on two different plasmids illustrated below the heatmap; the large plasmid carried *bla*$_{OXA-48}$. The accessory genome of the resistant and susceptible *Serratia marcescens* was compared using GenAPI (https://github.com/MigleSur/GenAPI) and confirmed by read mapping in Geneious Prime vR.9.

References

1. Gaio D, To J, Liu M, Monahan L, Anantanawat K, Darling AE. Hackflex: low cost Illumina sequencing library construction for high sample counts. bioRxiv. 2019;779215.
2. Seemann T. Prokka : rapid prokaryotic genome annotation. 2017;30(14):2068–9.
3. Wick RR, Judd LM, Gorrie CL, Holt KE. Unicycler: Resolving bacterial genome assemblies from short and long sequencing reads. PLoS Comput Biol. 2017;13(6):1–22.
4. Jolley KA, Maiden MCJ. BIGSdb: Scalable analysis of bacterial genome variation at the population level. BMC Bioinformatics. 2010;11.
5. Carattoli A, Zankari E, García-Fernández A, Voldby Larsen M, Lund O, Villa L, m.fl. In silico detection and typing of plasmids using PlasmidFinder and plasmid multilocus sequence typing. Antimicrob Agents Chemother. juli 2014;58(7):3895–903.
6. Camacho C, Coulouris G, Avagyan V, Ma N, Papadopoulos J, Bealer K, m.fl. BLAST+: architecture and applications. BMC Bioinformatics. 15. december 2009;10:421.
7. Lam MMC, Wick RR, Watts SC, Cerdeira LT, Wyres KL, Holt KE. A genomic surveillance framework and genotyping tool for Klebsiella pneumoniae and its related species complex. Nat Commun. 2021;12(1).
8. Treangen TJ, Ondov BD, Koren S, Phillippy AM. The Harvest suite for rapid core-genome alignment and visualization of thousands of intraspecific microbial genomes. Genome Biol. 2014;15(11):524.
9. Biedrzycka M, Izdebski R, Urbanowicz P, Polańska M, Hryniewicz W, Gniadkowski M, m.fl. MDR carbapenemase-producing *Klebsiella pneumoniae* of the hypervirulence-associated ST23 clone in Poland, 2009–19. J Antimicrob Chemother. 28. november 2022;77(12):3367–75.
